# Nanoscale rippling on polymer surfaces induced by AFM manipulation

**DOI:** 10.3762/bjnano.6.234

**Published:** 2015-12-02

**Authors:** Mario D’Acunto, Franco Dinelli, Pasqualantonio Pingue

**Affiliations:** 1 Istituto Struttura della Materia, ISM-CNR, via Fosso del Cavaliere 100, 00133 Rome; 2 Istituto di Scienza e Tecnologie dell’Informazione, ISTI-CNR, via Moruzzi, 1, 56124, Pisa, Italy; 3 Istituto Nazionale di Ottica, INO-CNR, via Moruzzi 1, 56124, Pisa, Italy; 4 Laboratorio NEST, Scuola Normale Superiore and Istituto Nanoscienze-CNR, Piazza San Silvestro 12, 56127 Pisa, Italy

**Keywords:** atomic force microscopy (AFM), films, nanomanipulation, nanomechanics, polymers, ripples

## Abstract

Nanoscale rippling induced by an atomic force microscope (AFM) tip can be observed after performing one or many scans over the same area on a range of materials, namely ionic salts, metals, and semiconductors. However, it is for the case of polymer films that this phenomenon has been widely explored and studied. Due to the possibility of varying and controlling various parameters, this phenomenon has recently gained a great interest for some technological applications. The advent of AFM cantilevers with integrated heaters has promoted further advances in the field. An alternative method to heating up the tip is based on solvent-assisted viscoplastic deformations, where the ripples develop upon the application of a relatively low force to a solvent-rich film. An ensemble of AFM-based procedures can thus produce nanoripples on polymeric surfaces quickly, efficiently, and with an unprecedented order and control. However, even if nanorippling has been observed in various distinct modes and many theoretical models have been since proposed, a full understanding of this phenomenon is still far from being achieved. This review aims at summarizing the current state of the art in the perspective of achieving control over the rippling process on polymers at a nanoscale level.

## Introduction

On deforming surfaces that are subject to external perturbations, ripple patterns commonly form over a wide range of length scales. For instance, macro ripples with a periodicity from meters to several centimeters are created by the wind blowing on sandy deserts and seashores [1]. The same behavior can be obtained by sliding loads on unpaved roads, ski slopes and rail tracks. Similarly, ion-beam sputtering on metal or semiconductor substrates can produce ripples on the microscale and nanoscale. The first example, reported in the literature, showed that low energy ion erosion of glass surfaces could lead to the formation of self-organized periodic patterns [2]. Since then, very regular patterns have been fabricated with this technique on a variety of materials, such as metals, semiconductors, and insulators, demonstrating the universality of this process [3]. The periodicity of the patterns can be tuned by varying the energy of the ions and ranges from a few tens of nanometers up to a few micrometers, with ion beam energies ranging from 0.1 to 100 keV. In particular two types of patterns are observed: ripples oriented either parallel or perpendicular to the direction of the ion beam, depending on the angle of incidence. Ultrashort laser pulses have been also employed for micro- and nano-structuring of polymer, semiconductor and metal samples [4]. In this case the ripple periodicity is correlated to the laser wavelength, while the orientation is determined by the laser polarization direction but it is also correlated with the laser scan direction and velocity.

Finally, the advent of atomic force microscopy (AFM) has opened the possibility to study single contact asperity contacts [5]. Nanoscale ripples have been then observed after performing one or many scans over the same area on a wide range of materials, namely ionic salts, metals, and semiconductors [6–9]. Regarding some crystalline materials, D’Acunto [10–11] and Filippov et al. [12] have successfully reproduced the experimental data via computational methods. Nevertheless, it is for polymeric films that the ripple formation has been studied most extensively [13–19]. Ripple structures on polymers can be produced either by performing a single scan or many scans on the same area of the sample. One can employ a heated tip [20–22] or a standard tip, on annealed or solvent-rich polymer films [23–24]. The ripple formation has been found to depend on a variety of material properties such as the preparation method, the mean molar mass of the polymer, the degree of crystallinity, as well as on the scanning conditions, namely the applied force, the tip shape and size, and the relative velocity. A wide spectrum of polymers has been investigated including polystyrene (PS) [13,20], poly(methyl methacrylate) (PMMA) [25], poly(ethylene terephthalate) (PET) [23], poly(vinyl acetate) (PVAc) [26] and poly(ε-caprolactone) (PCL) [23].

Recently, we have reviewed wear occurring on polymeric surfaces and how it can be exploited in order to deduce the molecular properties of polymer films [27]. In this review, we wish to specifically focus on the controlled formation of ‘nanoripples’. This phenomenon is related to plastic deformation, which in general does not lead to the formation of debris. In particular it can be exploited in order to pattern films for nanotechnology applications. The review is organized in three sections. The first one is dedicated to the most relevant observations reported in the literature. The second one deals with the theoretical modeling developed in order to interpret this phenomenon and predict further useful characteristics. The final section describes the strategies adopted up to now in order to achieve a good control of the phenomenon itself.

## Review

### Part 1: Phenomenological observations

The deformation of polymer surfaces upon contact with a sharp object is a phenomenon well known before the invention of the AFM methods. In particular, it was studied at a macroscopic level by sliding stiff objects, generally cones or spheres, over polymer samples. Plastic deformation and wear of polymer surfaces represented the subject of several experimental works carried out starting from the sixties of the last century. One of the most striking observations was the formation of macroscopic surface undulations, nowadays known as ‘Schallamach waves’ [28–29].

**Figure 1:**
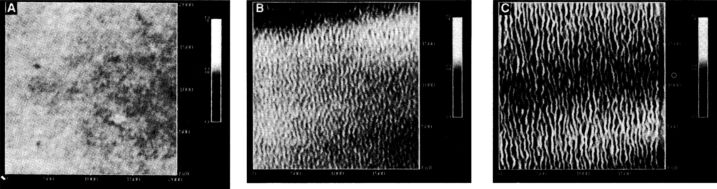
First experimental observations of the nanoripple formation on polymer films (2 × 2 μm^2^): from a pristine surface of PS (A), through the initial stages of deformation (B), to a fully developed pattern (C). The gray scale covers a height difference of 10 nm. Reprinted with permission from [14]. Copyright 1992 AAAS.

With the invention of AFM, the scientific interest moved to the investigation of plastic deformation and wear in polymer films on lower spatial scales. When one moves to smaller and smaller contact areas the wear features have dimensions well below the detection limit of optical microscopy, a typical instrument employed for the observations of Schallamach waves. Thus, grooves or ripples on the nanoscale can be imaged by means of AFM only after being formed with the same probe working in a lower load regime or in tapping mode. The first nanoripple observations have been reported when operating in the so called ‘contact mode’ (Figure 1), that is with the probe in contact and moved over a given area in a raster-like pattern. In this case the observed ripples were perpendicular or almost perpendicular to the fast scanning direction.

The researchers have soon realized that many parameters can affect the formation of nanoripples. These parameters depend on the characteristics of the tip–surface contact, the experimental conditions and the physico-chemical properties of the samples. Thus the dependences of nanoripple patterns on the scanning parameters have been extensively studied in the past decades. Equally, the dependences of nanoripple patterns on the properties of the samples have been qualitatively and, in some instances, quantitatively derived. The key observations and findings are reviewed in the following two sections.

#### Dependence on the scanning modality

**Scanning parameters:** Using an AFM, one can vary a range of experimental parameters such as the tip shape and surface chemistry, the applied load, the cantilever longitudinal and lateral stiffness, the scan direction and velocity, the spacing between successive lines (named ‘feeding’). Depending on these parameters, the nanoripple patterns form in either one or several scan frames. The most significant physical observables of the process are the lateral spacing and the vertical amplitude. In particular, they both tend to decrease with increasing the scan velocity [13]. On the contrary, both spacing and amplitude tend to increase with increasing the load applied and the number of scans [30].

**Single and multiple line scratching:** If one proceeds in a single line scratching mode, the nanoripple formation can be more easily controlled and determined than in a multiple line scratching mode [31–32]. In the latter case, the feeding and the number of scans over the same area are also extremely relevant. It has been reported that the patterns depend on the molecular weight (*M*_w_), on the scan direction and on the velocity [13]. In particular, as already stated above, the periodicity and the amplitude decrease with increasing the velocity. Ripple patterns can properly form only in the multiple lines scratching mode, sometimes immediately after the first passage of the probe. They are dependent on the probe movement direction, i.e., along parallel or slightly tilted (zig-zag) lines [15–16,31,33–35]. However, an analogy between the macroscale and microscale formations of ripples can be only drawn if the feeding is small enough that the whole scan can be described as the parallel movement of a number of tips moving together along the same direction, like a blade. The final pattern could also depend on the number of scans.

**Figure 2:**
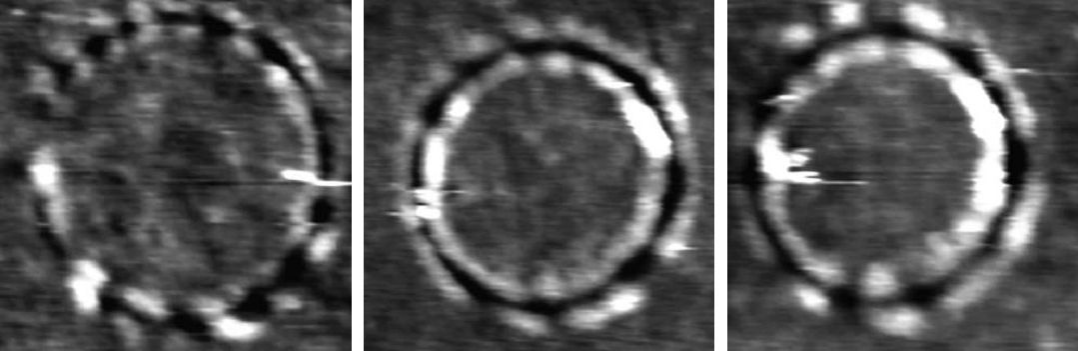
Topographical images in contact mode of three circular ripples created by a resistively heated AFM tip on a PPMA film. The image on the left is 410 × 410 nm^2^, the image in the middle is 445 × 445 nm^2^, and the image on the right is 375 × 375 nm^2^. All images have been obtained after only a few circular scans with a hot tip. The gray scale covers several nanometers. Reprinted with permission from [22]. Copyright 2009 American Physical Society.

**Tip trajectory:** Gnecco et al. have also evidenced that ripple patterns could be obtained via circular or spiral trajectories of the tip [22,36] inducing in this way the formation of a rippled structure along the circumference of a scanned circle (Figure 2). While scanning a PMMA surface with a minimum feedback, the authors have been able to record instantaneous variations in the cantilever vertical displacement. They have thus demonstrated that the ripples move after consecutive frames in a manner that can be considered similar to a wave packet travels in space. Additionally, they have managed to calculate the corresponding group velocity.

#### Dependence on material properties

**Figure 3:**
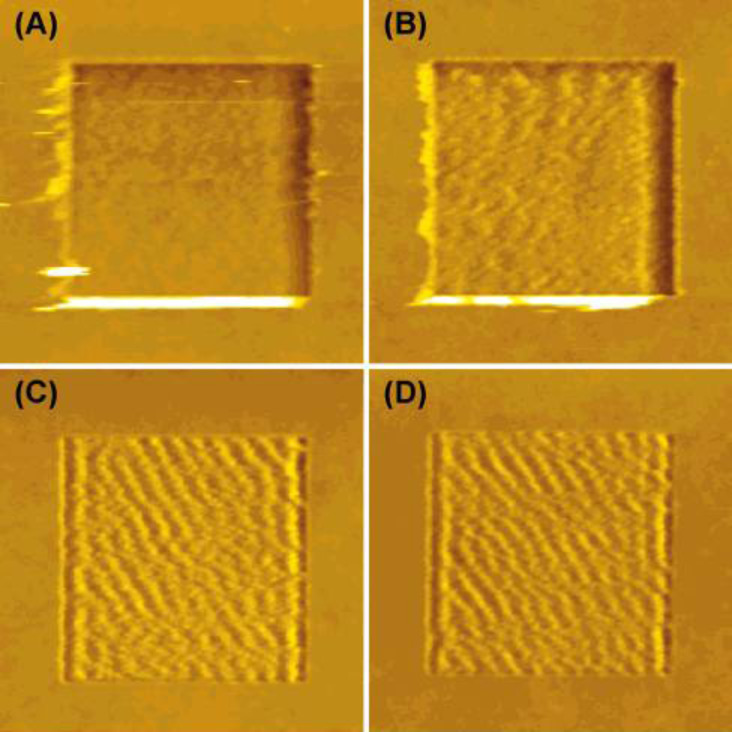
Topographical images (1.5 × 1.5 μm^2^) of surfaces scratched in the multiple line mode. The PS films analyzed have different *M*_w_ values: (A) 8 kDa, (B) 15.8 kDa, (C) 58 kDa, and (D) 164 kDa. The applied load is constant and equal to 10 nN. Adapted with permission from [31]. Copyright 2001 American Chemical Society.

**Molecular weight (*M*_w_):** For amorphous polymers, two relevant parameters are *M*_w_ and the monodispersity index. The viscoplastic behavior of samples made with different *M*_w_ and monodispersity index values can be drastically different. In particular the propensity for the formation of nanoripples varies enormously. This is clearly visible in the case reported in Figure 3. It has been suggested that this behavior is correlated to the critical *M*_w_ (*M*_c_) [37]. For *M*_w_ < *M*_c_, the molecules are never entangled [38]. The patterns and their load dependence reveal that the ripples either do not form or they are very irregular in shape and periodicity. In addition, bunches of molecules are moved and/or disrupted in an abrasive way (Figure 3A,B). On the contrary, for *M*_w_ > *M*_c_, the molecules are fully entangled, when the sample can be considered close to a thermodynamic stable state [38]. This is valid provided thermal annealing has been performed and solvent has been removed. Consequently, nanoripples form more easily, because the molecules cannot be removed from the surface but simply locally displaced (Figure 3C,D). For *M*_w_ close to *M*_c_ (approx. 30 kDa for PS) [38], it is possible to switch from a non-wear regime to abrasive wear by increasing the applied load or the number of scans.

In the work of Y. Sun et al. [13] the interpretation of the ripple patterning dependence on *M*_w_ is well depicted in terms of small and large sizes of the polymer molecules. The authors found well-ordered ripple patterns forming on PS films with a *M*_w_ of 250 kDa. No ripple formation was obtained for films with *M*_w_ values of 1.3 and 13 kDa, while operating in the same load and scan velocity conditions.

**Crystallinity:** Compared to amorphous regions, crystalline parts are generally less prone to wear. The most extensive and comprehensive studies of this topic have been carried out by Beake et al. [39–41]. They have considered the case of PET films, as this material can crystallize and samples can be made with different ratios of crystalline to amorphous components. The films were thus produced with the goal to obtain different sizes and densities of crystalline domains, either from standard molding or by means of applying uni- or bi-axial stretch to the melt when cooled down. These results show that the periodicity of nanoripples depends on the applied load value. The authors also found that the applied load needed to form ripples is higher for densely crystalline samples than for completely amorphous ones. Specific areas that initially show different morphologies give origins to different pattern periodicity. This behaviour is interpreted with different degrees of crystallization. In general, it can be suggested that for crystalline films one needs to initially create an amorphous layer and only then the ripples can fully form. Obviously, the whole process depends on how the amorphous layer is created, as the density and molecular conformations in such a layer are quite different from amorphous films. However, the *M*_w_ value of these samples is rarely known, making the interpretation of the data more complicated.

**Figure 4:**
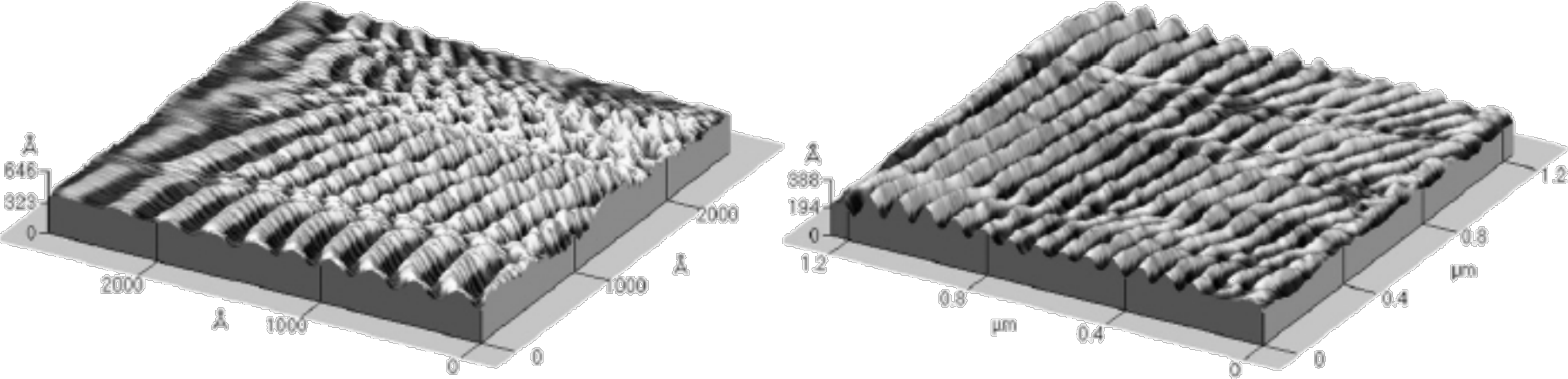
Solvent-enriched polymer films. Examples of 3D ripple structures on PCL (left) and PET (right). The images were obtained in air (relative humidity 40%), with a silicon conical shape tip with a nominal radius of 10 nm, a cantilever stiffness of 0.24 N/m, an applied load in the range between 2 and 10 nN, and scan velocity of 0.25 μm/s on 80 nm thick films. Reproduced with permission from [23]. Copyright 2007 Elsevier.

**Presence of solvents:** As it is widely known, polymers dissolve in solvents with similar chemo-physical properties. This is why they can be easily molded in a variety of shapes including thin films, by means of spin coating or drop casting. The relative presence of residual solvent molecules is responsible for weakening the mechanical properties. This phenomenon is known by the name of ‘plasticization’ [42]. It depends on the fact that solvent molecules are intercalated into the polymer molecules and the final result is a film swelling depending on the amount of solvent. Via a thermal post treatment the solvent can be finally removed, however the process depends on several parameters such as temperature, time, and solvent employed. Therefore assessing the amount and the type of solvent present is very crucial for the rippling process [13,15,23–24,43]. In Figure 4, we report an example of PCL and PET films exposed to different environmental conditions including solvent vapours. It is found that when these films deform faster and at lower load values compared to standard conditions. D’Acunto et al. [23] and Napolitano et al. [24] have reported that very regular ripple patterns can be created by means of a small applied load and just in a single scan on the surface of solvent-containing poly(ethylene terephthalate) (PET) films. In these works, solvent enrichment was obtained by simply avoiding the step of polymer curing and using the polymer film ‘as is’ after the spin coating deposition step. In this way, part of the solvent remains trapped in the polymeric thin film and the specific patterning procedure employed leads to the production of well-ordered ripple structures.

**Temperature dependence:** The temperature (*T*) dependence of nanorippling can be successfully addressed via AFM investigations. This has been done either through heating the probe [22,44] or the sample [19–20,45–47]. It is in fact commonly known that amorphous polymers have a second order thermodynamic discontinuity, named ‘glass transition’. This transition can be observed to various extents in the bulk as well as in the films depending on the crystalline degree of the analyzed sample. The value, at which it typically occurs, is called glass transition temperature (*T*_g_). In general, an AFM cannot observe the first order transition, which corresponds to crystalline melting.

**Figure 5:**
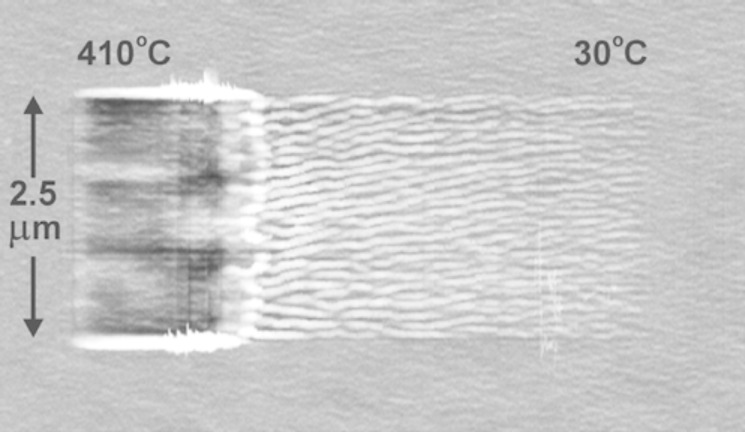
The worn area is the result of scanning a given surface with a variably heated tip, increasing *T* from 30 (right) to 410 °C (left). The PS film has a *M*_w_ of 70 kDa. The scan velocity is 50 μm/s. The gray scale covers 17 nm. Reprinted with permission from [20]. Copyright 2003 American Chemical Society.

Herein, we report one of the many experiments that can be found in the literature [20]. In order to create the morphology reported in Figure 5, a heated tip was scanned over a PS film (*M*_w_ = 70 kDa, bulk *T*_g_ = 100 °C) from right to left while increasing *T* from 30 to 410 °C. The surface starts to deform into nanoripples already at 30 °C. The lateral spacing and the vertical amplitude increase with increasing *T*. The transition from rippling to pileup wear occurs in a narrow window at around 237 °C. Many more studies deal with the change of the mechanical properties of polymers as a function of *T*. Typically the samples are heated rather than the tip, as these setups are easier to build and to control. For instance, Schmidt et al. investigated the ripple patterns, as they were concerned with the dependence of the viscoelasticity on *T* [45].

Similarly, Rice et al. have investigated the *T* dependence of rippling for the thin lamellar microphases forming in polystyrene/poly(ethylene oxide) block copolymer (PS-*b*-PEO) films [48]. In particular via an analysis of the ripple patterns, they have deduced local thermochemical parameters such as the melting temperature of PEO, the *T*_g_ of PS, the specific heat of PS-*b*-PEO, the melting enthalpy of PEO, and the Helmholtz free energy for unfolding (and melting) of PEO.

**Figure 6:**
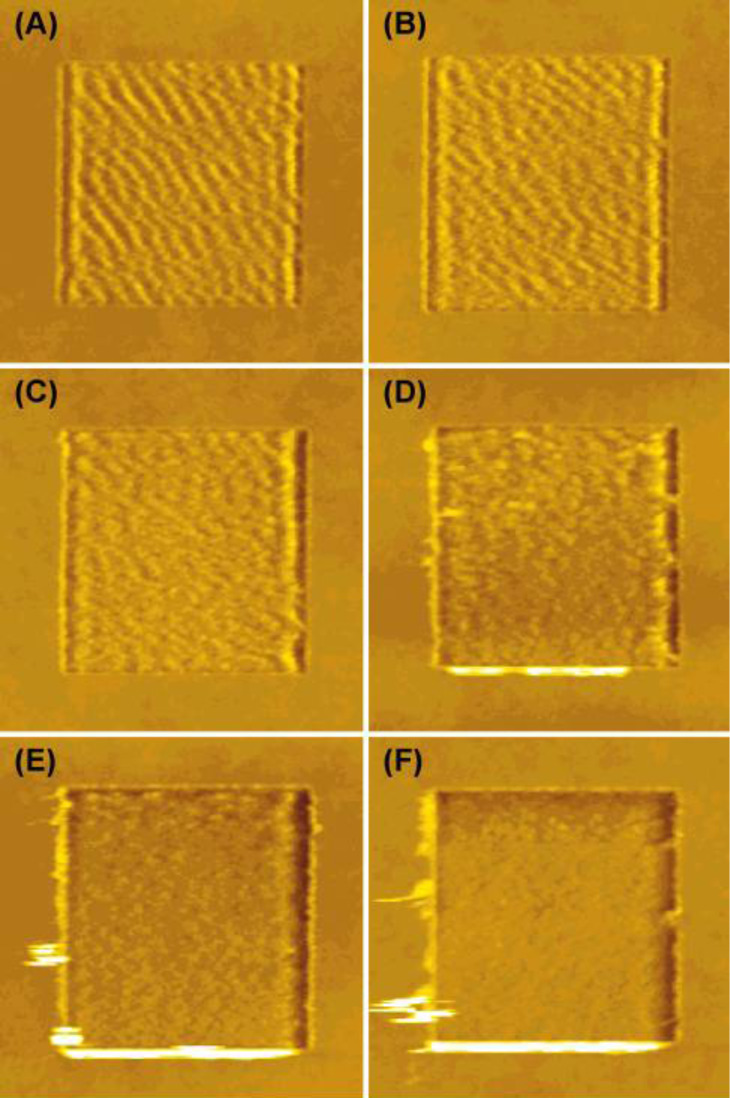
Topographical images (1.5 × 1.5 μm^2^) of patterns induced on films obtained from blending two PS solutions with *M*_w_ of 8 and 164 kDa. The ratio of the two solutions is (8 kDa/164 kDa): (A) 0/100, (B) 10/90, (C) 30/70, (D) 50/50, (E) 70/30, (F) 90/10, and (G) 100/0. The applied load is constant and equal to 10 nN. Reproduced with permission from [31]. Copyright 2001 American Chemical Society.

**Composite films:** Another class of samples showing a peculiar pattern formation are polymer blends. They can be miscible or immiscible, presenting clear phase separation or a similar morphology to homopolymers. In Figure 6 we show the case of films obtained from blending two PS solutions with *M*_w_ of 8 and 164 kDa in various proportions [31]. The *M*_w_ values were chosen to be above and below *M*_c_ for molecular entanglement. For the two extreme cases, wear patterns are equal to those shown in Figure 3. Other studies carried out on heteropolymer films can be also found in the literature. Buenviaje et al. have studied blends made of two immiscible components with different *T*_g_ values [49]. In samples exhibiting phase separation, regions corresponding to different materials would present nanoripples with different periodicities depending on the *T* value at which the experiment is carried out and with respect to the *T*_g_ values of the two components. Maas et al. [50] have conducted an experimental work on polysterene/polyvinylpyrrolidone (PS/PVP) copolymer films where the composition ratio is varied and the wear patterns reveal the different proportions of PS and PVP within the single molecules. Finally, Aoike et al. [51] have also reported studies on random copolymers.

### Part 2: Theoretical modelling

Once observed and described, like any other physical phenomenon, the formation of nanoripples should be rationalized so that it can be eventually controlled and exploited. In particular, nanorippling of polymer surfaces can be envisioned as a valid approach for lithographic purposes, particularly nowadays when nanotechnology is developing very quickly. However, achieving control of the ripple formation and therefore of polymer nanomanipulation requires a precise knowledge of all the parameters involved in the process. With the increasing number of observations, various models have been put forward to explain the occurrence of the nanopatterns. The main mechanisms proposed for nanopatterning induced by means of an AFM tip on polymer films are basically three: Schallamach waves [28], stick–slip behavior [52–53], and fracture-based descriptions [15,48].

On the macroscale, Schallamach waves are a reversible phenomenon that occurs at the surface of an elastomer when it slides past a stiff surface under compressive loads. It is attributed to buckling, i.e., waves of detachment driven by a tangential stress gradient along the contact zone of the sliding interface due to the breaking of adhesive bonds between the two surfaces [28]. On the nanoscale, however, ripples do not relax to their initial smooth shape when the tip is withdrawn and the sample is left unperturbed. Furthermore, the formation of the Schallamach waves on the macroscale was found to be dependent on a peeling phenomenon within the whole area of contact. The ripple periodicity on the nanoscale is found to be much larger than the actual contact area. Or, it is better to say that the possible formation of Schallamach waves within the contact area cannot be observed.

Therefore the formation of nanoripples is a phenomenon that occurs at the front edge of the contact. In particular, it has been suggested to be due to a stick–slip motion of the tip during the stage movement. A hole forms where the tip resides and a mound forms in front of the tip hindering the sliding motion [20]. The tip can slip over when the cantilever exerts a lateral force larger than the tip–sample adhesive interaction. Then the tip forms a new pair of hole and mound, so on and so forth. In order to form a continuous front, the positions of the mounds formed along adjacent lines need to be correlated and in phase. Notwithstanding the experimental differences, according to Aoike et al. [51], the friction coefficients measured in the macroscale and nanoscale cases are equivalent, when one normalizes the normal load values to the contact area. From this observation, they infer that the macroscale and nanoscale processes and their respective plastic deformations are determined by similar mechanisms.

Finally, Elkaakour et al. [15] have proposed another mechanism: A peeling process with the material pushed ahead of the contact via crack propagation. For an injection-molded polycarbonate surface, Iwata et al. [18] have found that the bundles are less stiff than the undamaged surface, which they have also interpreted as evidence of the presence of micro voids or cracks in the damaged region. Further experiments have been consequently stimulated pointing in this direction. It was argued that the crack formation might increase the volume of the perturbed region. This is a consequence of the mass conservation principle, assuming the incompressibility of the film. However, Rice et al. [48] did not observe the presence of peeling effects on locally heated PS-*b*-PEO copolymers. They suggested instead a vertical “crack” that may open once the tip sticks.

**Figure 7:**
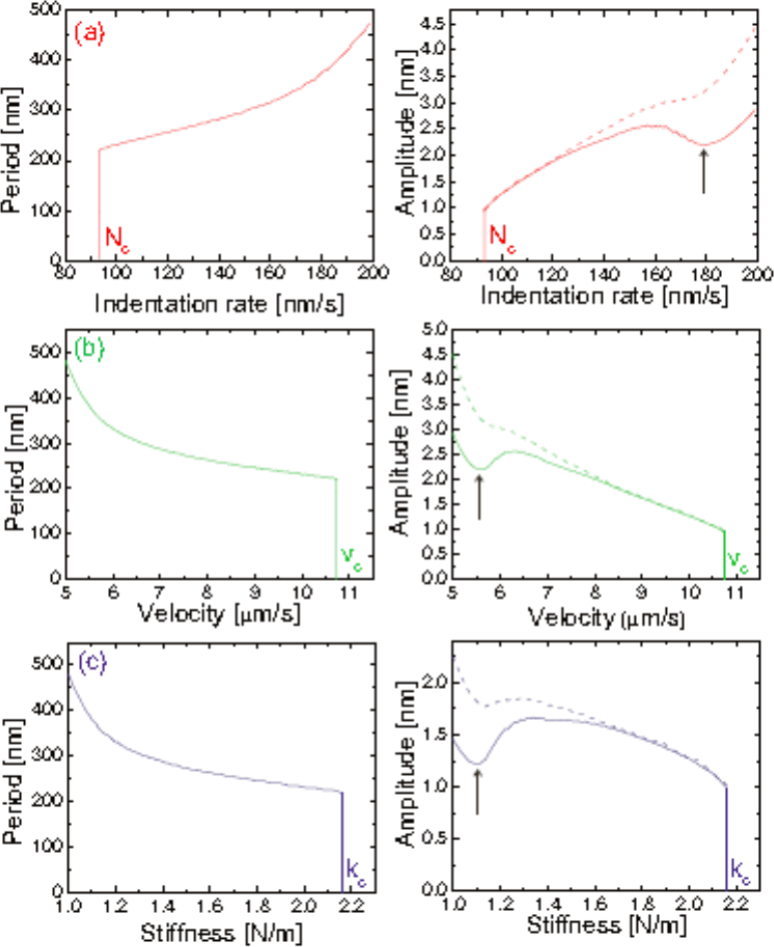
Ripple periodicity (left column) and ripple amplitude (right column, continuous curves) as a function of (a) the indentation rate *N*, (b) the sliding velocity *v* and (c) the lateral stiffness *k* with the other parameters kept fixed at the same values. When critical values (labelled with ‘c’ and corresponding to the vertical lines) are reached, the ripple pattern suddenly disappears. In the right column the maximum height (dashed curves) of the surface profiles is also shown. The corrugation can even decrease in the regions indicated by the arrows. Reproduced from [54].

More recently, Gnecco et al. [54] have employed the Prandtl model [55] to describe the nanoripple evolution in single scratch tests. In this model, the atomic structure of the substrate is not considered. This makes the continuum model more suitable for polymeric materials that are amorphous and have fully entangled molecules, i.e., for *M*_w_ >> *M*_c_. They have also introduced an indentation rate *N*, varying upon penetration of the tip. If one knows the time dependence *N*(*t*) and the indenter width, the process is found to be governed only by the scan velocity *v* and the lateral stiffness *k*. Specifically, the amplitude *A* and ripple periodicity increase when *N* exceeds a critical value *N_c_* or, vice versa, when *k* or *v* fall below the critical values of *v_c_* and *k_c_*, respectively (Figure 7). A transition from stick–slip to gliding can be also predicted for an indentation rate below a critical value or, alternatively, for large values of the sliding velocity, the lateral stiffness or the tip width. It is suggested also that this approach might be used to describe the evolution of similar rippling processes, by simply employing a proper indentation law. This analytical model could be also useful in order to understand phenomena such as the rippling of unpaved roads, ski slopes and rail tracks. A model fully describing the nanoripple formation, independently of the material involved, is however not yet available.

### Part 3: Towards achieving control

Based on the phenomenological observations and the theoretical models described in the two previous sections, some strategies have been devised and implemented in order to control the patterning, namely using cantilevers with miniaturized and integrated heaters; enriching the film with solvent; creating boundary conditions via defined tip trajectories; applying localized electrostatic fields through the tip to induce mechanical instabilities or crosslinking. Some of those efforts are summarized in this section.

#### Heated tip

One way to produce fast ripple patterns is to employ a heated tip (HT) as polymer properties strongly depend on *T*, especially in the glass transition regime. Vettiger et al. [56] have exploited the relative ease of patterning polymers above *T*_g_ for write/read storage operation in a thin polymer medium, obtaining bit densities similar or significantly higher than those achieved with magnetic storage systems at that time. Gotsmann and Dürig [30] have reported the creation of regular ripple structures employing HT on a 20 nm thick PS film. These patterns present a periodicity of around 100 nm and amplitude saturating at 20 nm. In addition, the authors have calculated energy activation for PS polymer pattern at *T* close to but below *T*_g_ to be on the order of about 0.4 eV.

**Figure 8:**
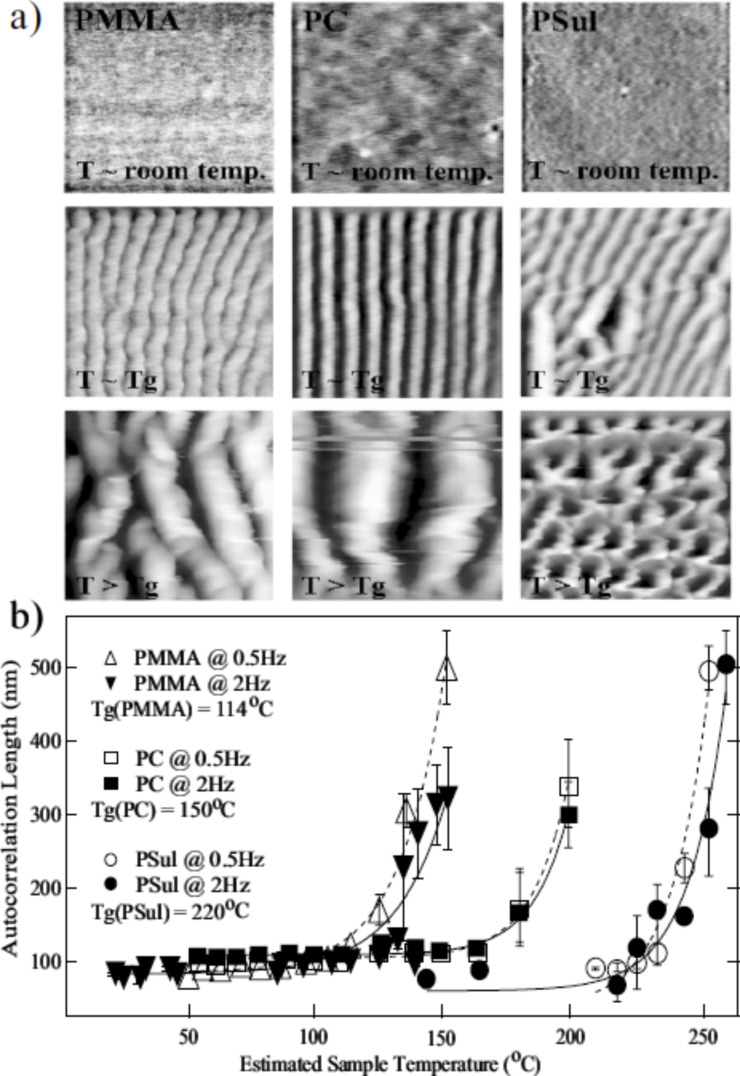
(a) Topographical images (1 × 1 μm^2^) of nanoripples created by means of a resistively heated tip at various *T* values on PMMA, PC and PS films. (b) A plot of the autocorrelation lengths versus estimated *T*, derived from the images shown in (a). It reveals an exponential dependence with increasing *T*. The two sets of data per material have been obtained for *v* of 0.5 μm/s (dashed lines) and 2 μm/s (solid lines). Reprinted with permission from [22]. Copyright 2009 American Physical Society.

Gnecco et al. [22] have reported that linear ripples with a period ranging from one to several hundreds of nanometers can be reproduced on the surface of polycarbonate (PC), poly(methyl methacrylate) (PMMA), and polystyrene (PS) films. Authors have clearly shown that the ripple formation varies with *T* and polymer type (Figure 8a). The dependence on *T* has been characterized and its behaviour correlated to the *T*_g_ value of the polymer investigated (Figure 8b). Such dependence can then be used to control the process. However, due to the exponential dependence on *T* of the ripple spacing (Figure 8), achieving a precise control over large spatial extensions is feasible but not easy.

#### Solvent enrichment

An alternative to fast patterning by means of HT is to scan solvent-enriched polymer films. Here, the solvent initially trapped in the vicinity of the surface has an analogous role to heating the tip. The film can be thus easily patterned under the passage of the AFM tip. Leach et al. [43] have obtained rippled PMMA surface (periodicity ≈ 100 nm) with a high normal force (180 nN) and a single scan using different solvents, such as water or alcohol–water mixtures.

**Figure 9:**
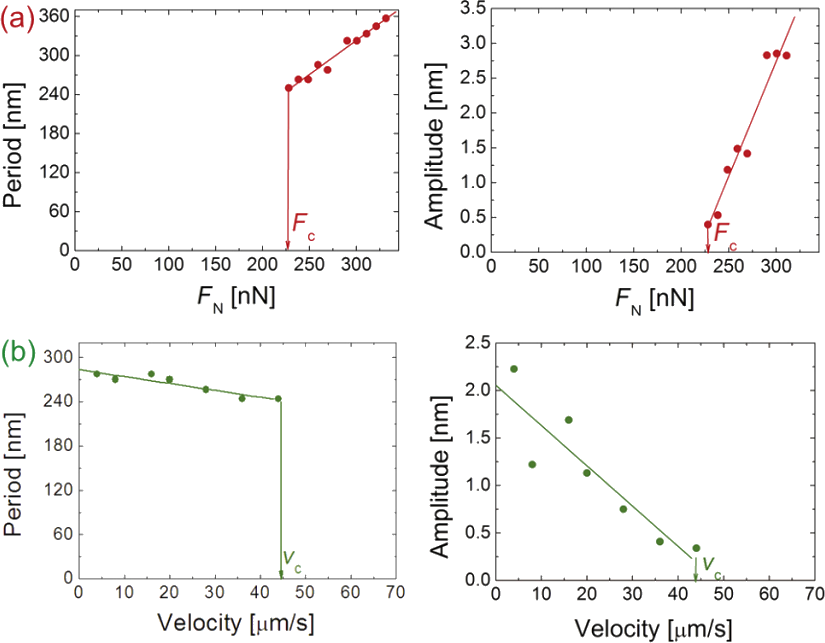
Theoretical variation of the ripple periodicity (left column) and ripple amplitude (right column) as a function of (a) the normal force *F*_N_ and (b) the sliding velocity *v*. Critical values, corresponding to the vertical lines, are labelled with ‘c’. The ripples are formed only if *F*_N_ > *F*_c_ ≈ 220 nN or if *v* < *v*_c_ ≈ 45 μm·s^−1^. Reproduced from [54].

**Figure 10:**
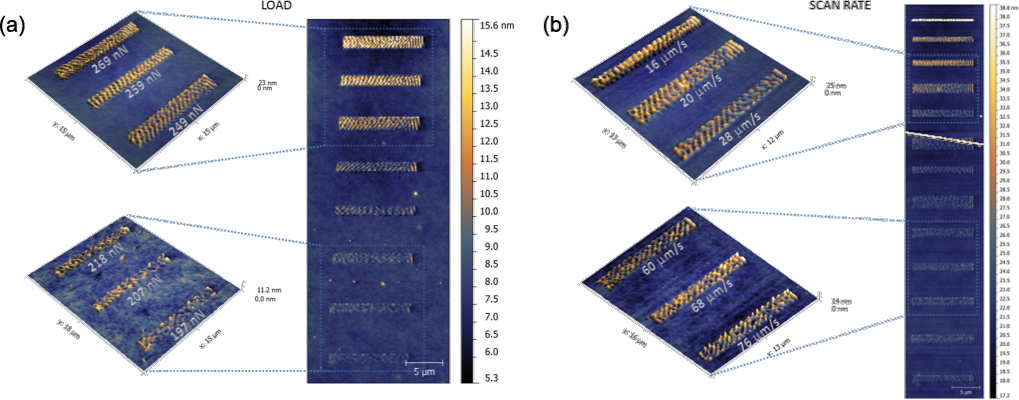
Topographical image of a series of scratches performed on 2 μm long lines on a PS film (*M*_w_ = 325 kDa), with increasing normal force *F*_N_ (a) and sliding velocity *v* (b). Unpublished data.

Gnecco et al. [54] have shown that surface ripples on a 400 nm thick PS film diluted with toluene can be explained by the competition between the driving spring force and the plastic response of the sample (Figure 7). The authors have firstly suggested that the ripples are expected to disappear when the indentation rate is below a critical value or the scan velocity is high (Figure 9), as more recently modeled. They verified the model on PS films enriched with toluene, demonstrating a good capability in controlling the periodicity and the amplitude of the pattern (Figure 10).

#### Influence of boundary conditions

To further improve the control when employing solvent-rich samples, we now show in this section an experiment exploiting a key observation of the AFM-based patterning: the correlation between the nanoripple orientation and the tip movement direction. The wearing properties of polymer surfaces can be thus exploited to create ripple structures by employing scans with proper boundary conditions that allow for the fabrication of self-assembled and ordered ripples.

**Figure 11:**
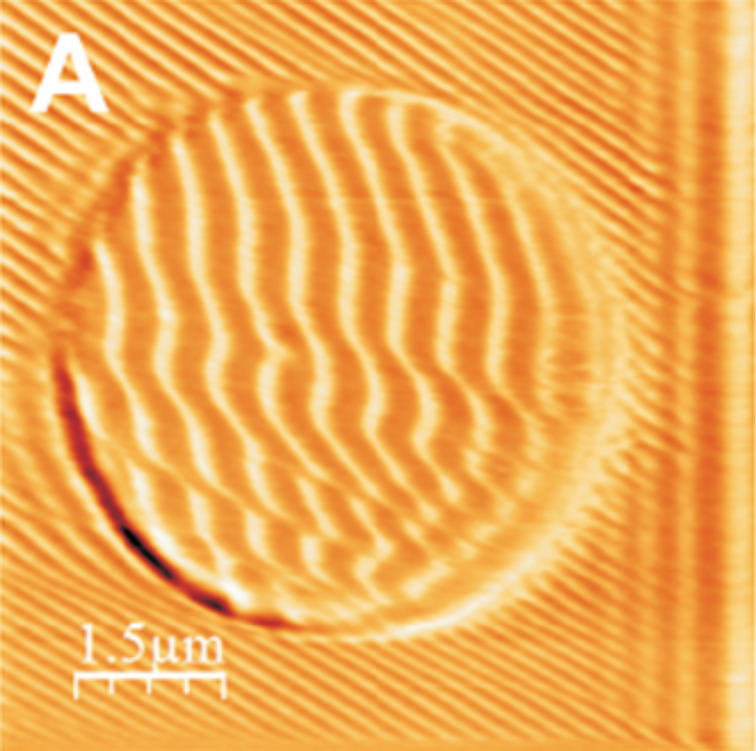
A topographical image of nanoripple patterns on a circular area. The color scale covers a range of 30 nm. The parallel lines on the right hand side are due to an instrumental artefact. Reproduced with permission from [24]. Copyright 2012 IOP Publishing.

Napolitano et al. [24] have carried out a series of experiments on PET films focusing their attention on the dependence of the ripple orientation on the boundary conditions. In particular, they have performed nanolithography by scanning the tip within circular, triangular, ellipsoidal and L-shaped areas. The results significantly show that the pattern orientation can be modified. In Figure 11, we report an example where, scanning the tip along parallel lines in a square region, the applied load is increased, compared to the rest of the area, only when moving in the central circular region.

#### High load regime: influence of scan angle, scan geometry and tip feed parameters

As reported above, most works on ripple patterning have been carried out by means of repeated scanning at small load values with a maximum of hundreds of nN. For manufacturing purposes, in order to improve the machining efficiency, it is however needed have precise periodical patterns fabricated in much shorter times. Thus some researchers have thought of increasing the load applied to the polymer films, studying the bundle formation mechanism at very high forces via a single scratch [57].

**Figure 12:**
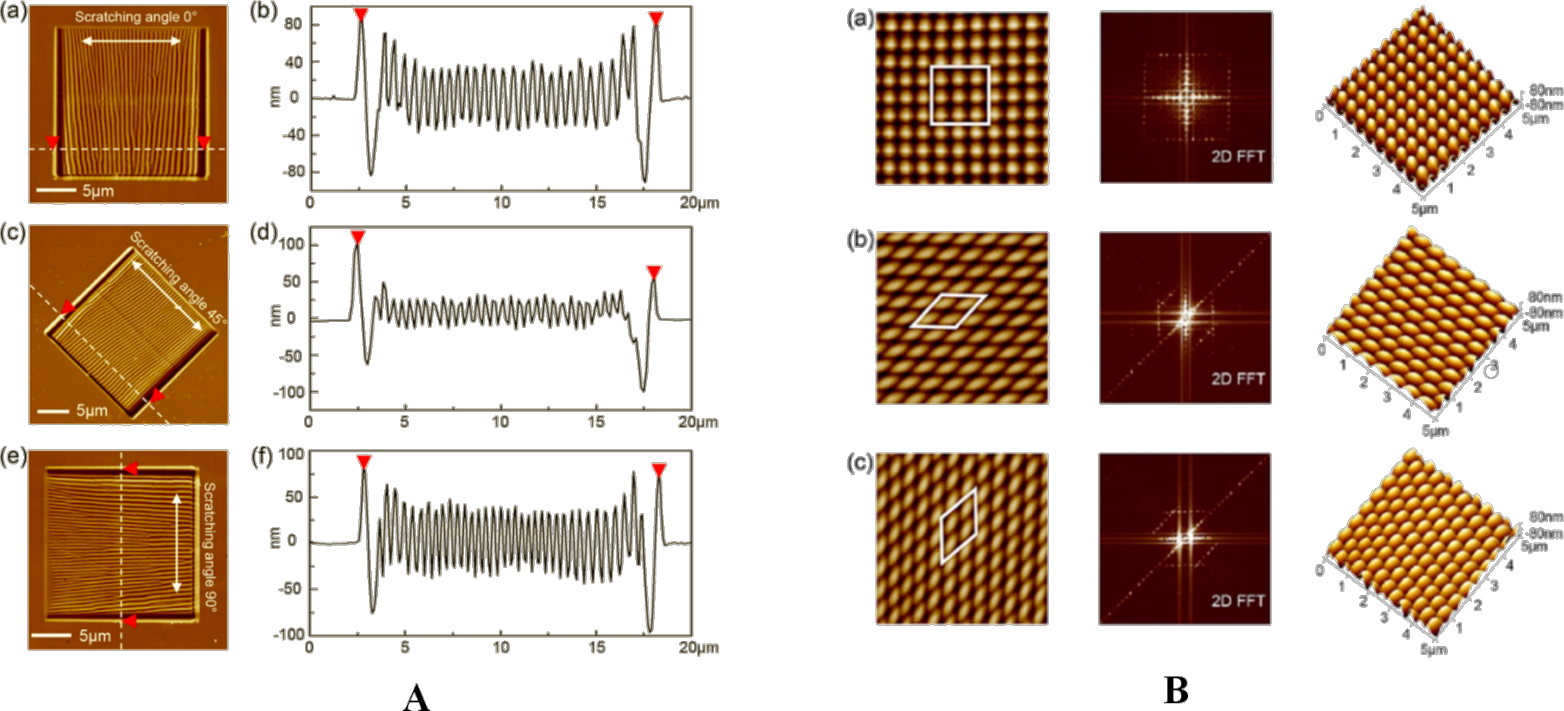
(A) The morphologies and cross-sections of the ripples. The corresponding scratching angles are 0° (a) and (b), 45° (c) and (d), and 90° (e) and (f). (B) Morphologies and 2D FFT images of 3D nanodot arrays. The scanning angles (a) 90° and 0°, (b) 90° and 45°, (c) and 0° and 45° of the two-step scratching method. Reproduced from [58].

Recently, Yan et al. [35] have improved the ripple patterning regularity and efficiency by scratching at very high loads. They have studied the influence of the scratch geometry and other parameter as the tip feed during the scan. The samples are injection-molded PC films purchased from a manufacturer. PC is known to be amorphous, however the *M*_w_ value is not reported. In another paper [58] the same research group has studied the effect of the scanning angle on the ripple formation by a single scratch at very high loads on polycarbonate (PC) (Figure 12). Finally, by combining scratching angles of 90 and 0°, 90 and 45°, and 0 and 45° in two-step machining, they have been able to fabricate an array of dot and diamond-dot structures with very controlled size and orientation, demonstrating in this way the capability, from a “nanomanipulation” point of view, of the AFM ripple patterning.

#### Electrostatic lithography

Finally, we briefly mention another family of techniques not strictly connected with wear mechanism but capable of producing a regular pattern and not necessarily ripple-like structures. These techniques rely on mechanisms based on the application of an electrostatic field to an AFM tip not in direct contact with the sample that may induce mechanical instabilities in a polymeric surface. One of such processes has been first discussed by Lyuksyutov et al. [59] and depends on the Joule effect. The polymeric volume under the tip can be heated locally at a *T > T*_g_ and attracted to the tip. Thus the film can be permanently deformed and instantly cooled once the voltage is switched off.

J. Y. Park et al. [60] show another possible approach where lines and dot arrays having nanoscale dimensions can be formed by electrochemical patterning. In this case, a film made of the precursor of polyterthiophene/poly(methyl methacrylate) (P3T/PMMA) copolymer can be cross-linked by the application of a voltage via the AFM tip. Specifically, the patterning was performed by means of an ultrasharp conductive tip with a radius of 20 nm applying voltages up to 10 V under ambient conditions (relative humidity: 50 to 60% and *T* = 22–23 °C).

#### How to avoid ripple formation

The nanoripple formation can be either a nuisance or an opportunity. In the framework of nanolithography, one should consider the development of tools for creating but also preventing the pattern formation. Regarding this second issue in the past it has been suggested, where applicable, to apply an out-of-plane ultrasonic vibration to the sample in order to avoid the plastic deformation of polymeric surfaces [61].

**Figure 13:**
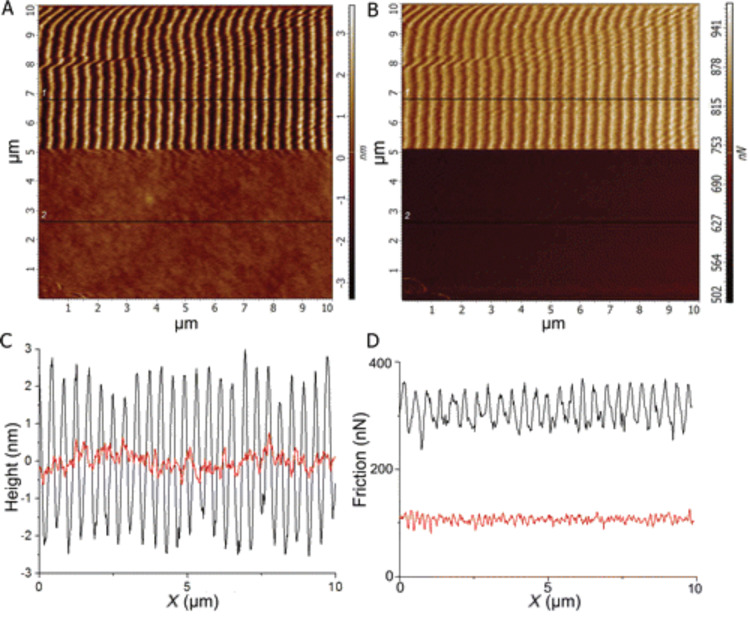
Topography (A) and lateral force (retrace, B) maps and profiles corresponding to the black lines (C and D) while scanning in contact mode (applied load *F*_N_ = 1378 nN) on a PS film. An AC voltage was applied to the transducer under the sample while acquiring the lower parts of the images, corresponding to a peak excitation amplitude of 0.55 nm (red profile). The drive voltage was zero while acquiring the upper parts of the image (black profile). Reprinted with permission from [63]. Copyright 2015 American Chemical Society.

The basic idea is to break the contact while scanning the tip before the mounds form and the lateral force builds up. More recently, (an example is reported in Figure 13) this principle has been successfully applied to avoid the nanoripple formation in polymeric films [62–63]. This demonstration represents an important achievement towards obtaining a AFM-based nanomanipulation tool that allows one to perform the ripple patterning only when it is desired.

## Conclusive remarks and future perspectives

In conclusion, the phenomenon of nanoripple formation on polymeric films via contact with AFM probes has been extensively studied in the past decades. The key parameters ruling this physical process have been clearly pointed out and described. Many theoretical works and models have been also proposed up to now. Some of them can reasonably describe and predict the nanoripple formation for several experimental conditions and for some specific material properties. This represents an important improvement for the nanomanipulation capabilities of the AFM technique when compared with initial purely phenomenological observations. An analytical model, fully and precisely describing the phenomenon of nanoscale rippling in polymers in all its complexities, has not been yet developed. However, it is likely that such a model would be analytically too complex and not easily applicable to each particular case. We hope, however, that this review will encourage further studies and future developments in understanding and control of the nanoscale rippling process on polymers.
